# Logistic regression trees for initial selection of interesting loci in case-control studies

**DOI:** 10.1186/1753-6561-1-s1-s57

**Published:** 2007-12-18

**Authors:** Radoslav Z Nickolov, Valentin B Milanov

**Affiliations:** 1 Department of Mathematics and Computer Science, Fayetteville State University, 1200 Murchison Road, Fayetteville, North Carolina 28301, USA

## Abstract

Modern genetic epidemiology faces the challenge of dealing with hundreds of thousands of genetic markers. The selection of a small initial subset of interesting markers for further investigation can greatly facilitate genetic studies. In this contribution we suggest the use of a logistic regression tree algorithm known as logistic tree with unbiased selection. Using the simulated data provided for Genetic Analysis Workshop 15, we show how this algorithm, with incorporation of multifactor dimensionality reduction method, can reduce an initial large pool of markers to a small set that includes the interesting markers with high probability.

## Background

One of the goals of genetic epidemiology is to identify genetic polymorphisms involved in the development of common diseases. Statistical methods for analyzing the relationship between a large number of candidate genetic loci and disease-related variables have been developed in genetic association studies. Studying complex diseases by means of single-locus methods may not be appropriate in the case of small main effects [1]. Methods to address these limitations have been developed. Multi-locus methods are specifically designed to find multiple disease loci that may influence the disease by intricate genetic patterns, gene × gene interaction and gene × environment interactions. These methods frequently find the best multilocus predictor using an exhaustive search, which makes it inapplicable to a large number of predictors.

To address this and other limitations, a flexible computational framework for detecting and interpreting gene × gene interactions has recently been proposed [2]. In its first step, entropy-based measures of information gain are used to select interesting predictors from the pool of possible thousands of candidates, a set of single-nucleotide polymorphisms (SNPs).

In this contribution, we present a method that could be used in the first step of the above-mentioned framework. We propose to use the LOTUS (logistic tree with unbiased selection) algorithm by Chan and Loh [3,4] in the initial selection of interesting predictors.

In our study of the utility of LOTUS for selecting a small number of interesting SNPs, we used simulated data from Genetic Analysis Workshop 15 (GAW15, Problem 3), with knowledge of the true model and simulation parameters used to generate the data.

We show that with LOTUS alone, and with incorporation of multifactor dimensionality reduction method (MDR) [5], an initial large set of SNPs can be reduced to a small set that includes the interesting markers with high probability.

## Methods

### Data set

The data set used was the GAW15 simulated rheumatoid arthritis (RA) data. We chose disease status for RA as the phenotype of interest. We used 50 replications of SNP data to create 50 samples of 1000 cases and 1000 controls. We created the cases by randomly sampling one affected member of each simulated family.

We did our analyses knowing the full model and simulation parameters used to generate the data. To explore the ability of LOTUS for selecting important predictors, we used some of the trait loci from the answers. For each sample, an initial set of 309 SNPs were considered, 303 of which were the simulated SNPs on chromosome 18. The rest were the trait loci A, B, C, D, E, and F. We used sample sizes of 250, 500, and 1000 cases (controls), respectively.

We also considered, as a more realistic scenario, the 303 SNPs of chromosome 18 only (trait locus E excluded). The sample size was 1000.

To estimate the false-positive rate, that is the probability of selecting a SNP not at causative locus, we used 4 of the 303 SNPs on chromosome 18.

### LOTUS

LOTUS (logistic tree with unbiased selection) is a method for automatic construction of logistic regression trees. LOTUS fits a piecewise (multiple or simple) linear logistic regression model by recursively partitioning the data and fitting a different logistic regression in each partition. This allows nonlinear features of the data to be modeled without requiring variable transformations. A few features make LOTUS especially appropriate for analysis and interpretation of large data sets: negligible bias in split variable selection, relatively fast training speed, applicability to quantitative and categorical variables, choice of multiple or simple linear logistic node models, and suitability for data sets with missing values.

LOTUS constructs logistic regression trees in a top-down fashion [3,4,6]. It deals with the selection bias problem due to some predictors taking more values than others, and distinguishes nonlinear from linear effects through the use of a Cochran-Armitage trend-adjusted chi-square test. It can fit either a multiple or simple logistic regression at each node. Once the initial tree is grown, it is pruned back using a pruning method similar to the classification and regression trees (CART) algorithm [7]. LOTUS uses deviance as the 'cost-complexity measure' instead of the sum of squared residuals. The tree with the lowest prediction deviance is chosen based on an independent test set or ten-fold cross-validation.

LOTUS allows the choice of one of three roles for each quantitative predictor variable: *f*-variable, for fitting only; *s*-variable, for splitting only; and *n*-variable for both splitting and fitting. In our application we treated each locus genotype as an *n*-variable. We fitted a multiple stepwise linear logistic regression tree. A *p*-value of 0.05 was used for forward selection and backward elimination. The maximum number of predictor variables to be selected at each node was chosen to be ten.

The LOTUS computer program is freely available [8].

### MDR

MDR is a nonparametric, combinatorial, model-free data-mining method, which has been successful in identifying gene × gene interactions in a balanced case-control design. With MDR, multilocus genotypes are pooled into high-risk and low-risk groups, thereby reducing the dimensionality of the genotype predictors from high dimensions to one dimension. That is, MDR employs constructive induction [9], the process of defining a new predictor as a function of two or more other predictors. The new one-dimensional multilocus-genotype predictor is used to choose the best set of loci from each one- to L-locus set according to classification and prediction errors. The MDR algorithm has reasonable power to detect epistasis [10].

### Selection of interesting SNPs

We studied two procedures for selecting a small set of interesting SNPs from an initial large set. In the first procedure we simply selected all SNPs in the final tree produced by LOTUS. However, if one selects as interesting only the SNPs in the final regression tree produced by LOTUS, some of the important SNPs, and possibly trait loci, might not be selected. Their effect might have been overlooked due to the strong effect of some of the selected loci, higher order interactions, or the parameter settings of the algorithm such as maximum number of predictors at each node. To address this, we incorporated MDR in our second selection procedure. We used MDR to select the best model among all possible one- to four-locus subsets of the predictor set selected by LOTUS. The markers in this best model were then removed from the initial set of SNPs and LOTUS was run again. The markers selected at the first and second runs of LOTUS constitute the final set of interesting SNPs.

Our second procedure may lead to a larger final set of interesting SNPs and thereby increased false-positive rate. However, the incorporation of MDR might improve the selection of important SNPs. Our goal is not to miss markers possibly involved in disease etiology. Although the proposed procedures are for initial selection and the false-positive rate is not of major concern, to keep it reasonably low we use at most two LOTUS runs.

LOTUS can process many thousands of predictors at one run. However, due to the computational limitations of MDR, we considered only hundreds of SNPs in our simulations, and chose the parameters of LOTUS accordingly.

## Results

We counted how many times in the 50 replications each of the trait loci was selected in the final set of interesting SNPs by each of the two procedures. First, we considered all 309 SNPs (303 of them on chromosome 18 plus the trait loci A, B, C, D, E, and F) as our initial set. Table 1 summarizes our results. Trait loci C and E were selected in the set of interesting SNPs in all 50 replications, in all cases considered. The selection rate of loci A, B, D, and F increased with the sample size for both procedures. It is easy to see that incorporating MDR in the selection process (our second procedure) significantly improved the selection rate of trait loci A, B, D, and F. For example, the selection rate of trait locus A increased from 26% to 30% when the sample size was 250, from 52% to 86% when the sample size was 500, and from 82% to 98% when the sample size was 1000.

**Table 1:** Selection rates for individual trait loci; 50 replications

	Selection rate (%)		
	
Trait locus	250 cases	500 cases	1000 cases
A	26 (56)^a^	52 (86)	82 (98)
B	20 (38)	58 (68)	68 (86)
C	100 (100)	100 (100)	100 (100)
D	76 (96)	98 (100)	100 (100)
E	100 (100)	100 (100)	100 (100)
F	88 (98)	100 (100)	100 (100)

^a^The numbers in parentheses are the selection rates for Procedure 2.

Next, we considered chromosome 18 SNPs only: 303 SNPs, excluding the trait locus E. The sample size was 1000. SNP-269 that is physically closest to the trait locus E (and possibly in high linkage disequilibrium with it) was selected 92% of the time by the first procedure and 98% of the time by the second procedure. In the cases when SNP-269 was not selected by the first procedure, the set of interesting SNPs included SNP-268, which is also flanking the trait locus E.

To estimate the false-positive rate we considered four randomly chosen SNPs on chromosome 18, SNP-44, SNP-58, SNP-119, and SNP-127, which are not at the causative locus E. We counted how many times each SNP was selected by the two procedures in the final set of SNPs. Table 2 summarizes our results. The false-positive rates for our first procedure were close to 5%, the nominal level used by LOTUS for forward selection and backward elimination. For the second procedure the false-positive rates almost doubled. This was expected, because our second procedure combines the selected sets from two LOTUS runs.

**Table 2:** False positive rates for individual loci; 50 replications

	False positive rate (%)		
	
Locus	250 cases	500 cases	1000 cases
SNP-44	0 (0)^a^	6 (8)	6 (8)
SNP-58	2 (4)	6 (10)	0 (2)
SNP-119	4 (8)	2 (4)	2 (2)
SNP-127	2 (8)	2 (4)	2 (2)

^a^The numbers in parentheses are the false-positive rates for Procedure 2.

## Conclusion

Our analysis of the GAW15 simulated data demonstrates the usefulness of the logistic tree with unbiased selection algorithm (LOTUS) [3,4] for reducing the number of SNPs in a case-control study by selecting a small number of interesting SNPs from among a large number of candidate loci. This algorithm may be used in the first step of the computational framework proposed by Moore et al. [2] or in any other multi-stage procedure. Applying LOTUS to a set of 309 SNPs (6 of them trait loci), we found that the trait loci were selected in the set of interesting SNPs with a high rate when either the loci effect or the sample size is large.

We want to point out that in most of our simulations LOTUS finished with a trivial tree, and the number of SNPs selected as interesting was less than 20. If the initial set is very large, one can increase the number of maximum predictor variables to be selected at each node.

In our study we included only genetic markers as predictors. However, LOTUS can use any categorical (gender, smoking status, etc.) or quantitative predictors, which may improve the selection process. This is the object of our future studies.

## Competing interests

The author(s) declare that they have no competing interests.
